# Making a giant: Uncovering gene regulation underlying the formation of giant cells in Arabidopsis sepals

**DOI:** 10.1093/plcell/koad066

**Published:** 2023-03-05

**Authors:** Humberto Herrera-Ubaldo

**Affiliations:** Assistant Features Editor, The Plant Cell, American Society of Plant Biologists, USA; Department of Plant Sciences, University of Cambridge, Cambridge CB2 3EA, UK

A plant is composed of millions of cells that receive spatial and temporal cues during cell identity specification (reviewed in Xu et al. 2021). Cell identity must be regulated to coordinate specific functions and for the development of distinctive anatomical features. Cis-level regulatory elements such as enhancers regulate cell type identity (reviewed in Long et al. 2016). Giant cells establish sepal curvature and are involved in plant defense (Roeder et al. 2012). In this issue of *The Plant Cell*, **Lilan Hong, Byron Rusnak, Clint S. Ko, and colleagues** (Hong et al. 2023) uncovered the regulatory mechanism driving cell-specific gene expression during giant cell formation in Arabidopsis sepals. In particular, they dissected the function of a 1 kilobase region previously shown to enhance gene expression in giant cells (Roeder et al. 2012).

To investigate how this region directs expression specifically in giant cells, the authors divided it into four regions, R1 through R4. The four regions were used alone or combined into two- or three-unit chimeras to generate fluorescent reporter lines. The R1 + R4 or R1 + R3 line conferred giant cell-specific expression; R2, R2 + R3, or R2 + R4 exhibited broad epidermal expression patterns, and R1 or R4 alone resulted in no expression. These results indicate that the R1 region directs expression towards the giant cells, whereas the R2 region is involved in broad epidermal pattern expression (see Figure).

**Figure.**
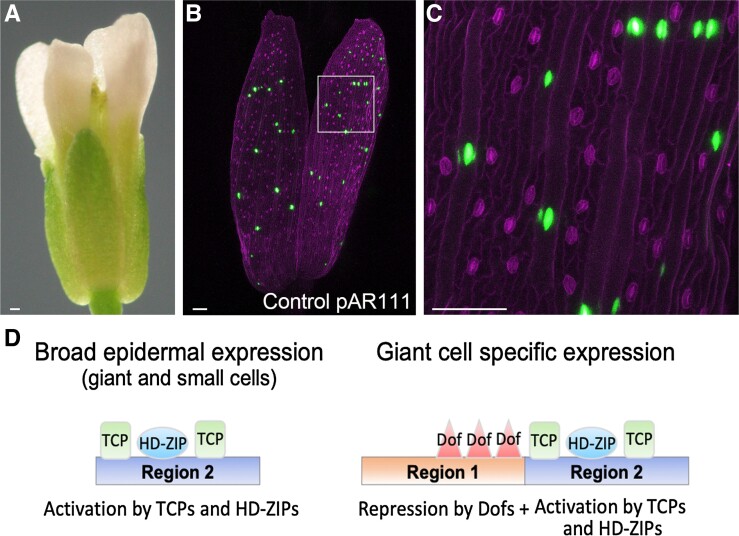
An enhancer drives giant cell gene expression. **A)** Overview of the Arabidopsis flower. **B)** and **C)** Venus signal in the giant cells’ nuclei in the sepals. The pAR111 marker contains the full-length giant cell enhancer driving the expression of 3xVenus-N7. **D)** A model for the cell type specificity in the giant cell enhancer involving two key regions and three families of transcription factors driving activation/repression. Scale bars, 100 μm. Adapted from Hong et al. (2023), Figures 5 and 6.

To identify trans-factors acting on R1 and R2 to regulate enhancer activity, the authors conducted a yeast 1-hybrid (Y1H) assay with nearly all the transcription factors in Arabidopsis. Of the 150 identified interactors, proteins belonging to the Class II CINCINNATA-like TCP (TCP2, TCP3, TCP4, and TCP10) interacted with R2 and a 100 base pair (bp) junction sequence between R1 and R2. To functionally validate the protein–DNA interactions, the authors crossed the *jaw-1D* mutant (exhibits downregulated TPC expression) with the complete enhancer or the R2 reporter lines. In the full enhancer and R2 reporter lines, the downregulation of the TCPs did not affect the formation of giant cells but displayed a reduced signal. Two putative TCP binding motifs were found in the R2 region. Electrophoretic mobility shift assay (EMSA) confirmed that TCP4 binds to the R2 region. Additionally, mutations of the TCP binding motifs in the complete enhancer or R2 reporter lines caused a near-total loss of the reporter signal. In summary, the TCP protein binding to R2 activates expression driven by the enhancer.

The authors hypothesized that regulators binding to the R1 region confer expression specificity to the giant cells. In the initial Y1H assays, several DNA-binding one zinc finger (Dof) proteins [AtDOF2.2, HIGH CAMBIAL ACTIVITY 2 (HCA2), and AtDOF5.8] interacted with the R1 and the 100 bp junction region which possess three Dof TF binding motifs. The addition of the Dof binding motifs to Region 2 restricted its expression in the giant cells. On the other hand, the deletion of the Dof binding motifs expanded and displayed an epidermal expression pattern. Overexpression of *DOF2.2*, *HCA2*, or *DOF5.8* reduced the expression of the enhancer in the giant cells and presented altered sepal development. These results demonstrate the role of R1 and Dofs in reducing the expression driven by the enhancer.

Y1H results revealed another TF family, HD ZIP, that binds to the R2 and the 100 bp junction sequence. Previous studies identified some HD ZIP proteins in Arabidopsis, such as HOMEODOMAIN GLABROUS 11 (HDG11) and MERISTEM LAYER 1 (ATML1), as regulators of giant cell identity (Roeder et al. 2012). The authors overexpressed *ATML1* and found ectopic giant cells in the sepals, suggesting a role in the activation of enhancer activity. EMSA and mutational analyses of reporter lines verified that ATML1 binds to the HD ZIP binding motif in R2, and the enhancer activity is reduced when the binding is disrupted. The authors generated reporter lines with varying levels of *ATML1* expression (using knockout and overexpression lines, Meyer et al. 2017) and observed increased signal intensity with increased levels of ATML1, indicating the function of this HD ZIP protein as an activator of gene expression. The model suggests that the combined effect of TCPs and ATML1 binding to R2 and Dof binding to R1 drives giant cell specificity.

In summary, this study illustrates how complex mechanisms control gene expression underlying cell-type specification and cell-specific gene expression. In this case, the formation of giant cells depends on the activation by TCP and HD ZIP transcription factors combined with the repression activity of Dof transcription factors in different regions.
